# Supplementary material for: Three to Tango: Inhibitory Effect of Quercetin and Apigenin on Acetylcholinesterase, Amyloid-β Aggregation and Acetylcholinesterase-Amyloid Interaction
Source: Pharmaceutics. 2022 Oct 30;14(11):2342. doi: 10.3390/pharmaceutics14112342 (PMC9699245; doi:10.3390/pharmaceutics14112342)

Supplementary Materials

Three to Tango: Inhibitory Effect of Quercetin and Apigenin on Acetylcholinesterase, Amyloid-β Aggregation and Acetylcholinesterase-Amyloid Interaction

Irene Álvarez-Berbel[1,‡], Alba Espargaró[1,‡], Antonio Viayna[2,‡], Ana B. Caballero[3], Maria Antònia Busquets[1], Patrick Gamez[3,4], Francisco Javier Luque[2,*], Raimon Sabate[1,*]

1 Department of Pharmacy and Pharmaceutical Technology and Physical-chemistry, School of Pharmacy, and Institute of Nanoscience and Nanotechnology (IN2UB), University of Barcelona, 08028 Barcelona, Spain
2 Department of Nutrition, Food Sciences, and Gastronomy, School of Pharmacy and Institute of Biomedicine, Campus Torribera, University of Barcelona, Prat de la Riba 171, E-08921, Santa Coloma de Gramenet, Spain
2 Department of Inorganic and Organic Chemistry, Faculty of Chemistry, University of Barcelona, 08028 Barcelona, Catalonia, Spain; Institute of Nanoscience and Nanotechnology (IN2UB) and NanoBIC
2 Catalan Institution for Research and Advanced Studies, Passeig Lluís Companys 23, 08010 Barcelona, Catalonia, Spain
* Correspondence: rsabate@ub.edu (RS), fjluque@ub.edu (FJL)
‡ Irene Álvarez-Berbel, Alba Espargaró and Antonio Viayna contributed equally to this work

Figure S1. Representation of the conformational variability found for residues Tyr337 in the catalytic site, and Trp286 in the peripheral site, as found in the X-ray structures 4M0F, 6CQU, and 6O4X. The protein backbone is shown as a gray cartoon. View along the axis that leads from the peripheral site to the catalytic site along the gorge.

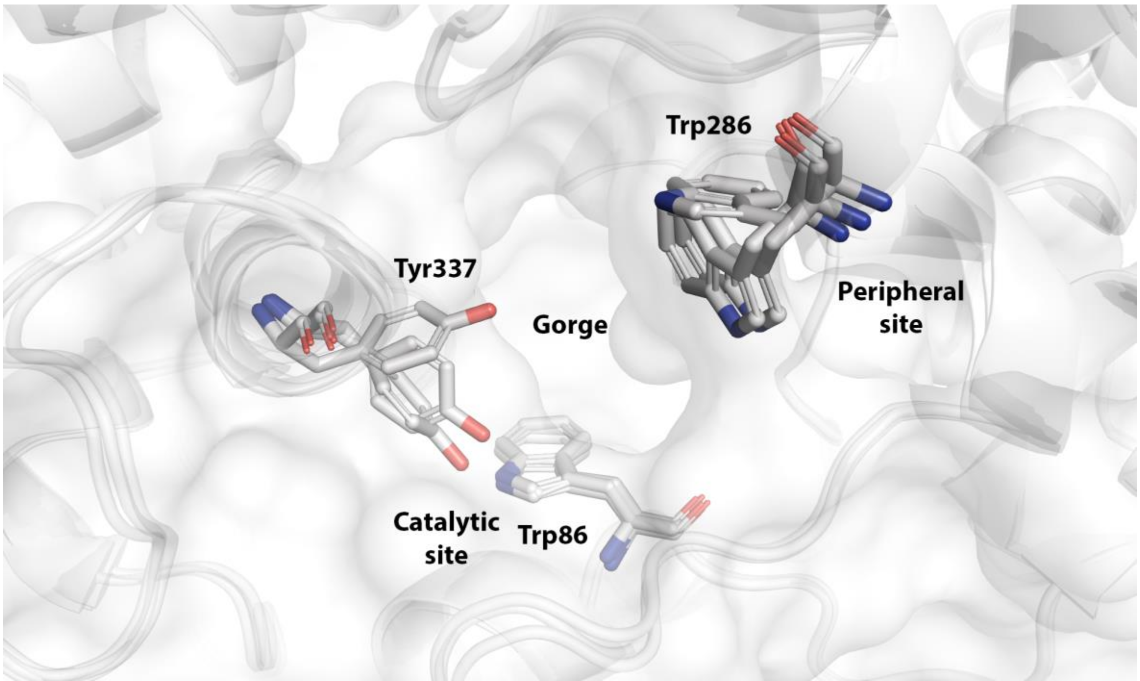

Figure S2. AChE preferential interaction determined by SDS-PAGE experiments. Supernatant fraction of Aβ40 aggregation induced by AChE in the presence and absence of the flavonoids.

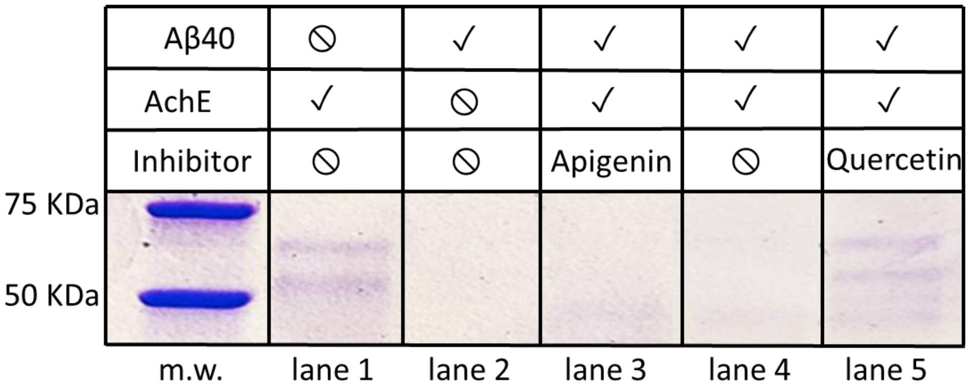

Figure S3. AChE activity. **a**) Time evolution of the absorbance at 412 nm for different AChE concentrations. **b**) Activities of different AChE concentrations using the Elman's assay. **c**) Values of the slopes obtained for various AChE concentrations.

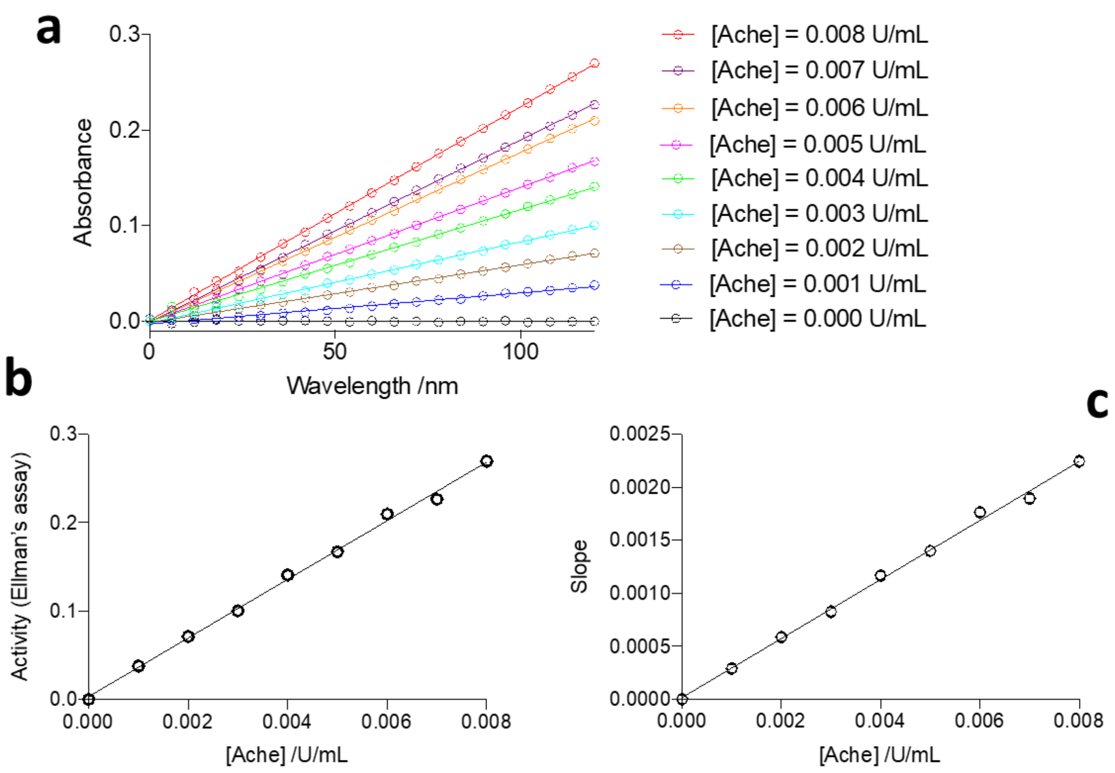

Figure S4. Quenching effect of (**a**) apigenin and (**b**) quercetin on the intrinsic AChE fluorescence. [AChE] = 0.02 μM; [Apigenin or quercetin] = 0 to 1 μM. λ_{exc} = 275 nm, λ_{em} = 332 nm.

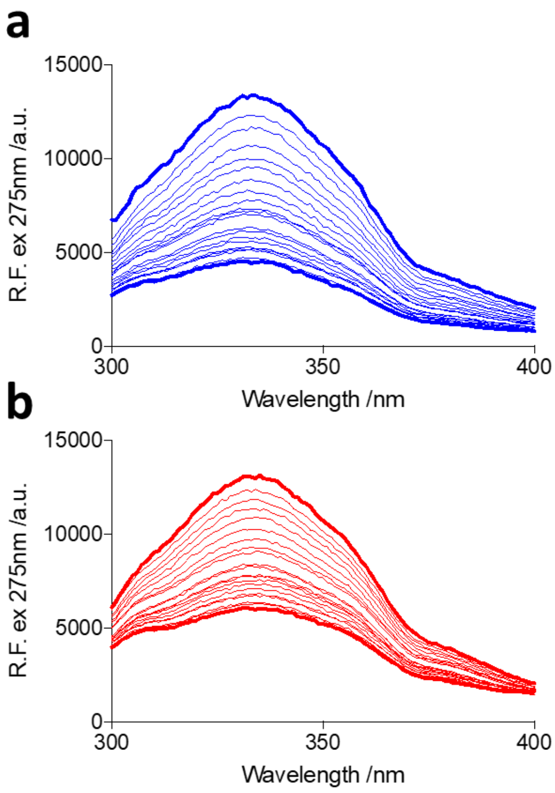

Figure S5. RMSD profile (light color) determined for the backbone atoms (in green), heavy atoms in the binding site (in red), and heavy atoms in the ligand (in blue) for simulations of AChE-quercetin complexes (values averaged every 50 snapshots are shown in dark color).

a) 4M0F (replica 3).

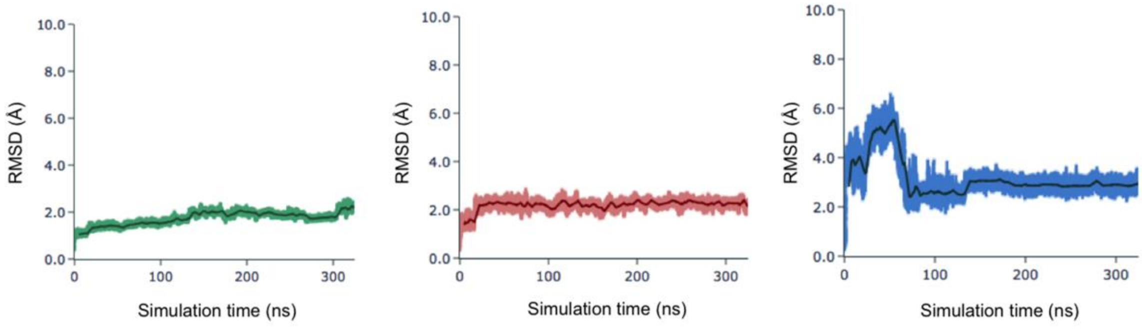

b) 4M0F (replica 2).

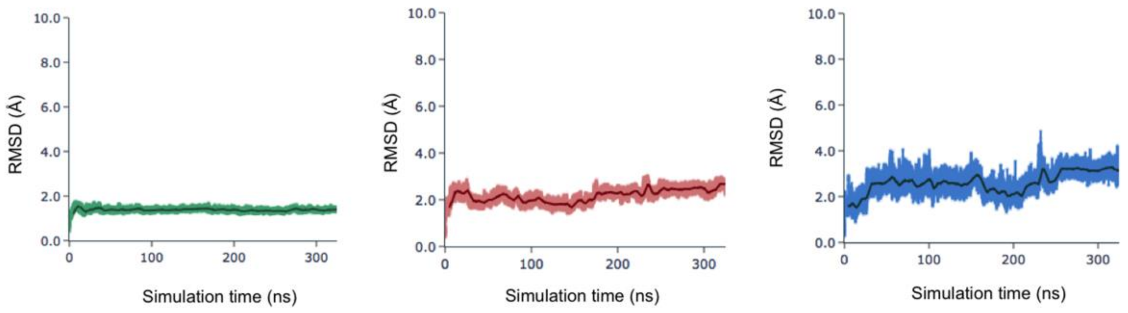

c) 6CQU (replica 1).

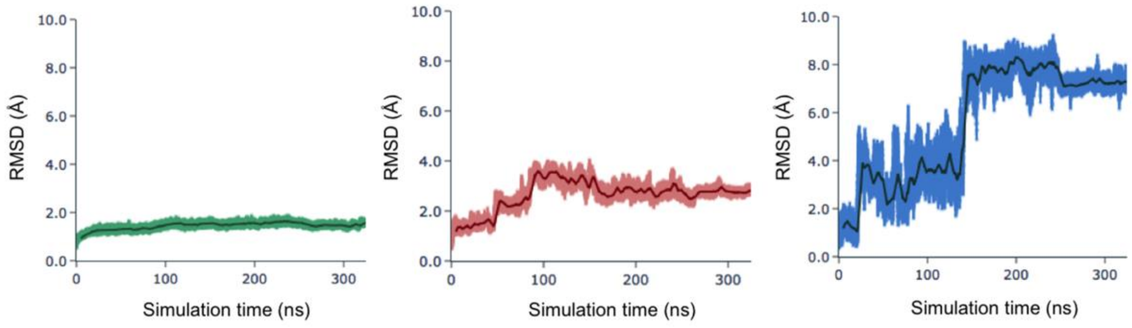

d) 6CQU (replica 2).

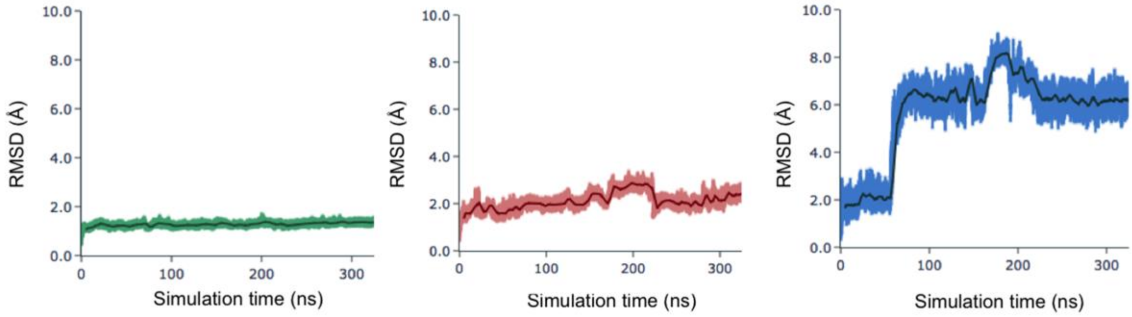

e) 6O4X (replica 3).

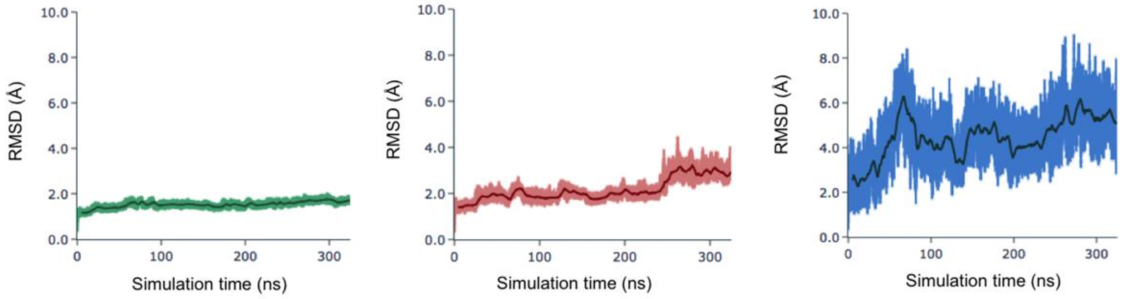

Supplement: Supplementary file 1 [file pharmaceutics-14-02342-s001.zip › pharmaceutics-1989344-supplementary.pdf]
